# Comparative analysis of left atrial size and appendage morphology in paroxysmal and persistent atrial fibrillation patients

**DOI:** 10.1002/joa3.13224

**Published:** 2025-01-23

**Authors:** J. Pongratz, L. Riess, S. Hartl, B. Brueck, C. Tesche, D. Olbrich, M. Wankerl, U. Dorwarth, E. Hoffmann, F. Straube

**Affiliations:** ^1^ Heart Center Munich‐Bogenhausen, Department of Cardiology and Internal Intensive Care Medicine Munich Hospital Bogenhausen, Munich Municipal Hospital Group Munich Germany; ^2^ Department of Electrophysiology Alfried Krupp Hospital Essen Germany; ^3^ Department of Medicine Witten/Herdecke University Witten Germany; ^4^ Kardiologie Praxis Erkelenz Erkelenz Germany; ^5^ Department of Cardiology Clinic Augustinum Munich Munich Germany; ^6^ Department of Radiology, Neuroradiology and Nuclear Medicine Munich Hospital Bogenhausen, Munich Municipal Hospital Group Munich Germany

**Keywords:** atrial fibrillation, catheter ablation, cryoballoon, left atrial appendage, left atrium

## Abstract

**Purpose:**

Pulmonary vein isolation (PVI) is effective in treating atrial fibrillation (AF), but outcomes are worse for persistent AF (persAF) patients than paroxysmal AF (PAF) patients. The study aimed to identify differences in left atrial (LA) and left atrial appendage (LAA) anatomy in different AF types.

**Methods:**

In a single‐center observational study, a blinded retrospective analysis of preprocedural cardiac computed tomography angiography (CCTA) images was performed. The study evaluated the dimensions of the LA and pulmonary veins (PV), as well as the size and morphology of the LAA using a 3D electroanatomical mapping system.

**Results:**

Between 2012 and 2016, a total of 1103 patients underwent second‐generation cryoballoon PVI. Of these, 725 patients (65.7%) had CCTA available, and 473 of these (65.2%) had sufficient quality for measurements. The mean age of the patients was 66.3 ± 9.5 years, and PAF was present in 277 (58.6%) participants. The study found that in persAF patients, LA dimensions such as LA volume [mL] (108; 125; *p* < .001) or PV ostial dimensions were significantly larger than in those with PAF. LAA volume [mL] (8.3; 9.2; *p* = .005) and LAA ostial area [mm^2^] (325; 353; *p* = .01) were enlarged in persAF. There were no significant differences regarding LAA morphology, with the overall distribution being “windsock” (51%), “chicken‐wing” (20%), “cauliflower” (15%), and “cactus” (13%).

**Conclusion:**

Compared to PAF, persAF patients had significantly larger LA as well as LAA dimensions. LAA morphological types were distributed equally in both groups suggesting that LAA morphology may not be associated with the underlying AF type.

## Graphical abstract

This study shows that persAF patients exhibit larger LA and LAA dimensions compared to PAF patients, with no significant differences in LAA morphology. These findings suggest that preprocedural analysis of anatomical dimensions, rather than LAA shape, could play a more important role in guiding personalized ablation strategies, particularly for complex AF cases.

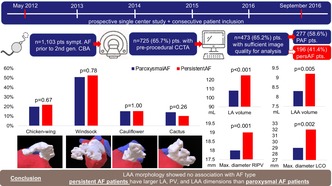

## 1 INTRODUCTION

Atrial fibrillation (AF) is a prevalent and sustained abnormal heart rhythm with significant clinical implications worldwide. It is highly correlated with an elevated risk of stroke, heart failure, and mortality.^1^ The primary cause of both paroxysmal atrial fibrillation (PAF) and persistent atrial fibrillation (persAF) is ectopic triggers that originate from the pulmonary veins (PV).^2^ Hence, the fundamental aspect of catheter ablation is to achieve pulmonary vein isolation (PVI) electrically. At present, there are two recommended techniques: (a) radiofrequency ablation (RFA) by point‐by‐point method or (b) single‐shot cryoballoon ablation (CBA).^2^ Nonetheless, particularly in patients with persAF, success rates of ablation are significantly lower in comparison to those with PAF.^3^ The reason for this disparity may be attributed to the progressive nature of AF, which induces functional and structural changes, leading to the formation of a vulnerable substrate for triggers and re‐entries outside of the pulmonary veins (PVs).^4^

Previous studies have identified various predictors of AF recurrence after ablation, such as LA volume, LAA volume, and mitral regurgitation.^5^ However, the anatomical differences between PAF and persAF patients, particularly concerning LA and LAA morphology, have not been thoroughly explored. Moreover, the benefit of preprocedural CCTA usage is still uncertain.

Therefore, the primary objective of this analysis was to evaluate the differences of anatomical features, with a special focus on LA and LAA anatomy, between PAF and persAF patients to better understand how they might influence ablation outcomes and provide insights into more personalized treatment strategies.

## 2 METHODS

### 2.1 Study design and objectives

This is a sub‐analysis of patients with PAF and persAF from a previously published observational single‐center study by Straube et al.^5^ PAF and persAF were defined according to current AF guidelines.^2^ Detailed information on the methods used, including preprocedural, intraprocedural, and postprocedural management, has been described before.^5^

The overall study population was divided into two groups: Group A comprised patients with PAF, and Group B comprised patients with persAF. Baseline characteristics and measurement parameters were subsequently compared.

### 2.2 Patient selection

This study included consecutive symptomatic AF patients scheduled for their initial ablation procedure by means of PVI. All patients were treated with the second‐generation cryoballoon (Arctic Front Advance™, Medtronic Inc., MN, USA) and prospectively enrolled in the institutional observational registry. Patients with a recent, high‐quality preprocedural CCTA that allowed assessment of LAA anatomy were considered for inclusion in the blinded analysis. The clinical indications for CCTA were primarily to exclude coronary artery disease (CAD) and secondarily to evaluate LA and PV anatomy before CBA. No patients were excluded from CBA based on variations in LA, PV, LAA anatomy, or LA volume as determined by CCTA. Exclusion criteria included long‐standing persistent AF, left atrial diameter >60 mm, intracardiac thrombi, severe valvular disease, advanced heart failure, and prior left atrial ablation.

### 2.3 Preprocedural imaging and anatomical assessment

Before the ablation procedure, all patients underwent transthoracic and transesophageal echocardiography to rule out the presence of left atrial thrombus. Additionally, the individual left atrial anatomy was assessed using a 64‐slice CT scanner (Brilliance 64, Philips Medical Systems, Cleveland, Ohio, USA), with retrospective ECG gating and 3D reconstruction. Scanning was performed at 120 kVp with an effective tube current of 600 mAs. The slice collimation was 64 × 0.625 mm, with a gantry rotation time of 0.4 s and a pitch of 0.2. Images were reconstructed with a slice thickness of 0.9 mm and 0.45 mm increments. Contrast enhancement was achieved with 80 mL of contrast agent (Imeron 400 MCT, Iomeprol 81.65 g/100 mL, Bracco, Konstanz, Germany) injected at a flow rate of 5 mL/s, followed by a 50‐mL saline flush. A blinded observer (JP) analyzed, segmented, and measured each CCTA image both visually and quantitatively, evaluating the defined parameters and the morphological classification of the LAA using EnSite Precision™ (Abbott Medical GmbH, Eschborn, Germany). Multiplan volume‐rendered postprocessing was used to create a 3D visualization. After anatomical segmentation of the pulmonary veins, left atrium, and LAA, all 2D and 3D measurements were performed.

### 2.4 Data acquisition, management, and quality control

**FIGURE 1:**
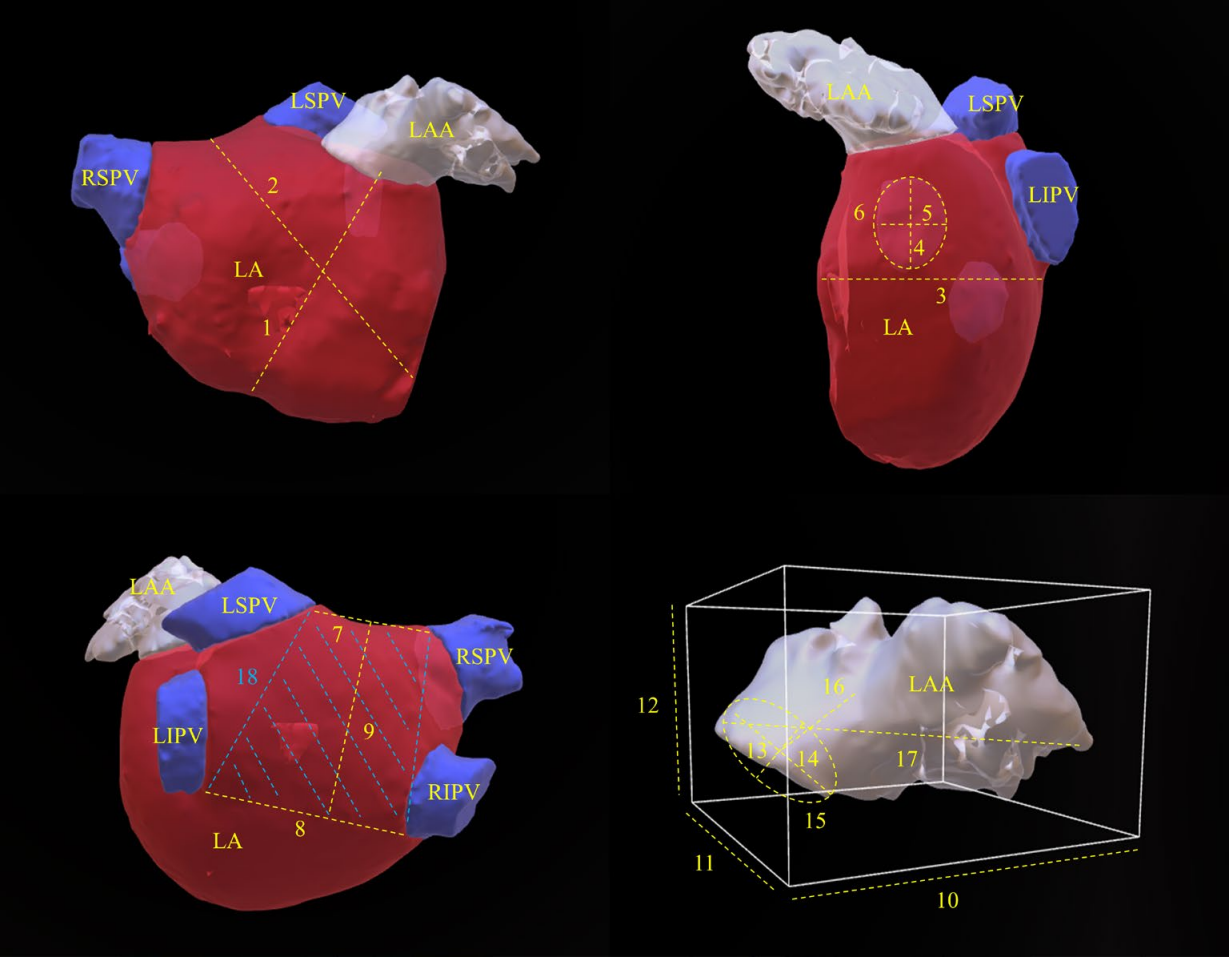
Demonstration of measurement data. The presented image depicts the most important dimensions of the LA and LAA in three‐dimensional reconstructed images obtained through CCTA. The numerical values labeled alongside the dotted lines denote distinct measurements. 1: septum‐orifice distance, 2: distance of the mitral valve annulus to the LA roof, 3: LA depth, 4: max. ostial diameter of the RSPV, 5: minimal ostial diameter of the RSPV, 6: area of the RSPV, 7: roof top line, 8: roof bottom line, 9: posterior wall box height, 10: maximal LAA width, 11: maximal LAA depth, 12: maximal LAA height, 13: maximal diameter of the LAA ostium, 14: minimal diameter of the LAA ostium, 15: area of the LAA ostium, 16: distance to the first bend, 17: max. LAA length, 18: posterior wall box area. LA, left atrium; LAA, left atrial appendage; LIPV, left inferior pulmonary vein; LSPV, left superior pulmonary vein; RIPV, right inferior pulmonary vein; RSPV, right superior pulmonary vein.

**FIGURE 2:**
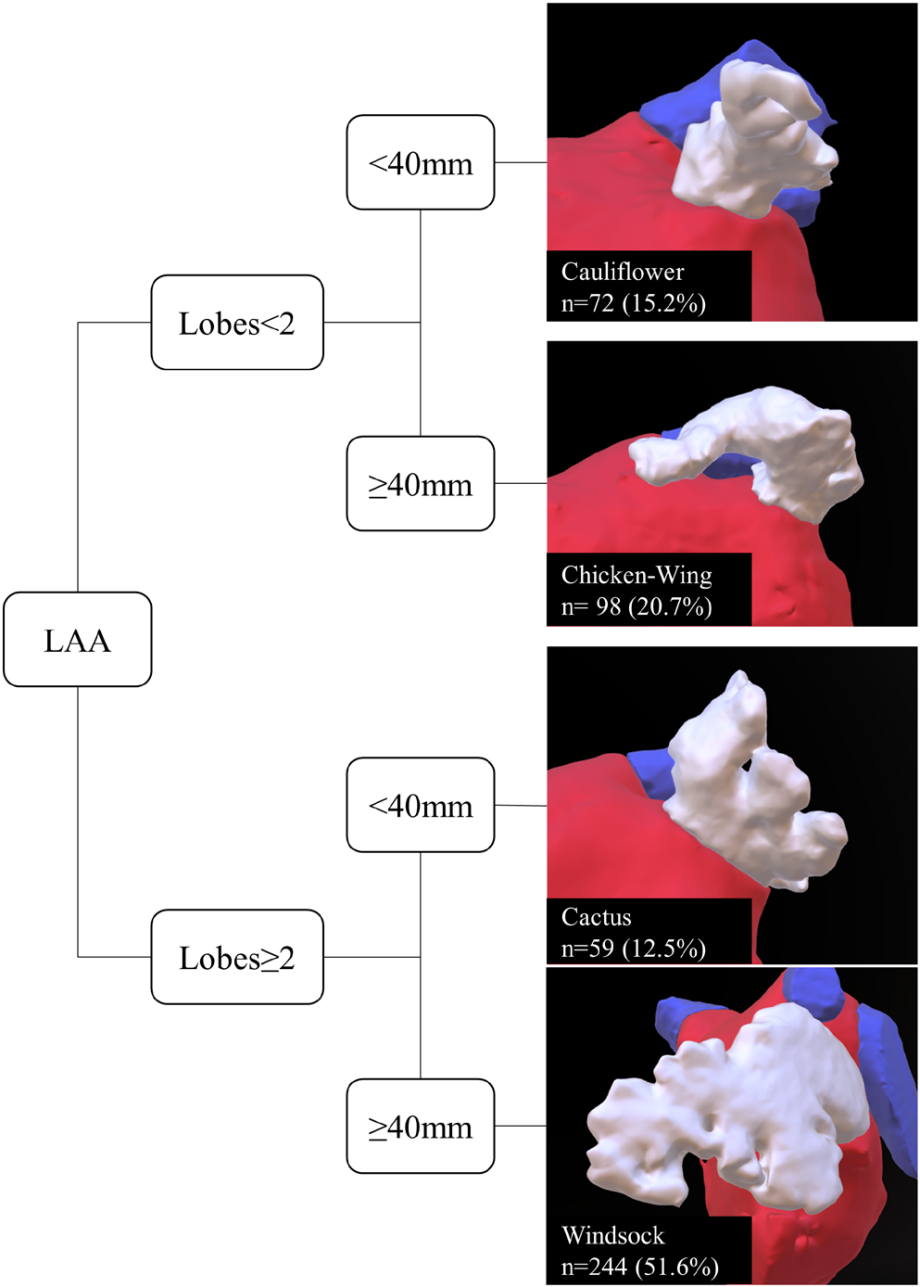
LAA morphology. The presented illustration portrays the various morphological types of the LAA as defined by Wang^6^ and characterized by Kimura's quantitative qualifiers.^7^ Based on the number of lobes and length of the LAA, it was categorized into four types: Windsock, chicken‐wing, cactus, and cauliflower. The predominant distribution of LAA types observed in the study is also presented. LAA, Left atrial appendage.

Baseline characteristics were obtained from institutional registry entries and individual medical records. The acquisition of measurement data from CCTA images was previously described in detail.^5^ The data evaluated included two‐dimensional (2D) measurements and three‐dimensional (3D) measurements obtained through multiplan volume‐rendered postprocessing. Most important measurement data are illustrated in Figure 1. Left atrial volume index (LAVI) and left atrial appendage volume index (LAAVI) were calculated using the DuBois formula. The morphological classification of the LAA was based on the criteria established by Wang et al. and Kimura et al. and divided into four types: chicken‐wing, windsock, cactus, and cauliflower (see Figure 2 for details).^6^, ^7^

### 2.5 Statistical analysis

The collected data was entered into Microsoft Excel 2016 (Microsoft Corp., Redmond, WA, USA) and then imported into SPSS version 25 (IBM Corp., Armonk, NY, USA) for statistical analysis. Categorical parameters were presented as numbers and percentages, while continuous variables were reported as means with standard deviations or medians with their respective interquartile range based on the Shapiro–Wilk test. The statistical analysis of categorical data was carried out by conducting the chi‐squared test, while the statistical analysis of continuous data involved the use of the Student's *t*‐test for normally distributed data and the Mann–Whitney *U* test for non‐normally distributed data. Statistical significance was defined as *p* ≤ .05.

## 3 RESULTS

### 3.1 Study population

**FIGURE 3:**
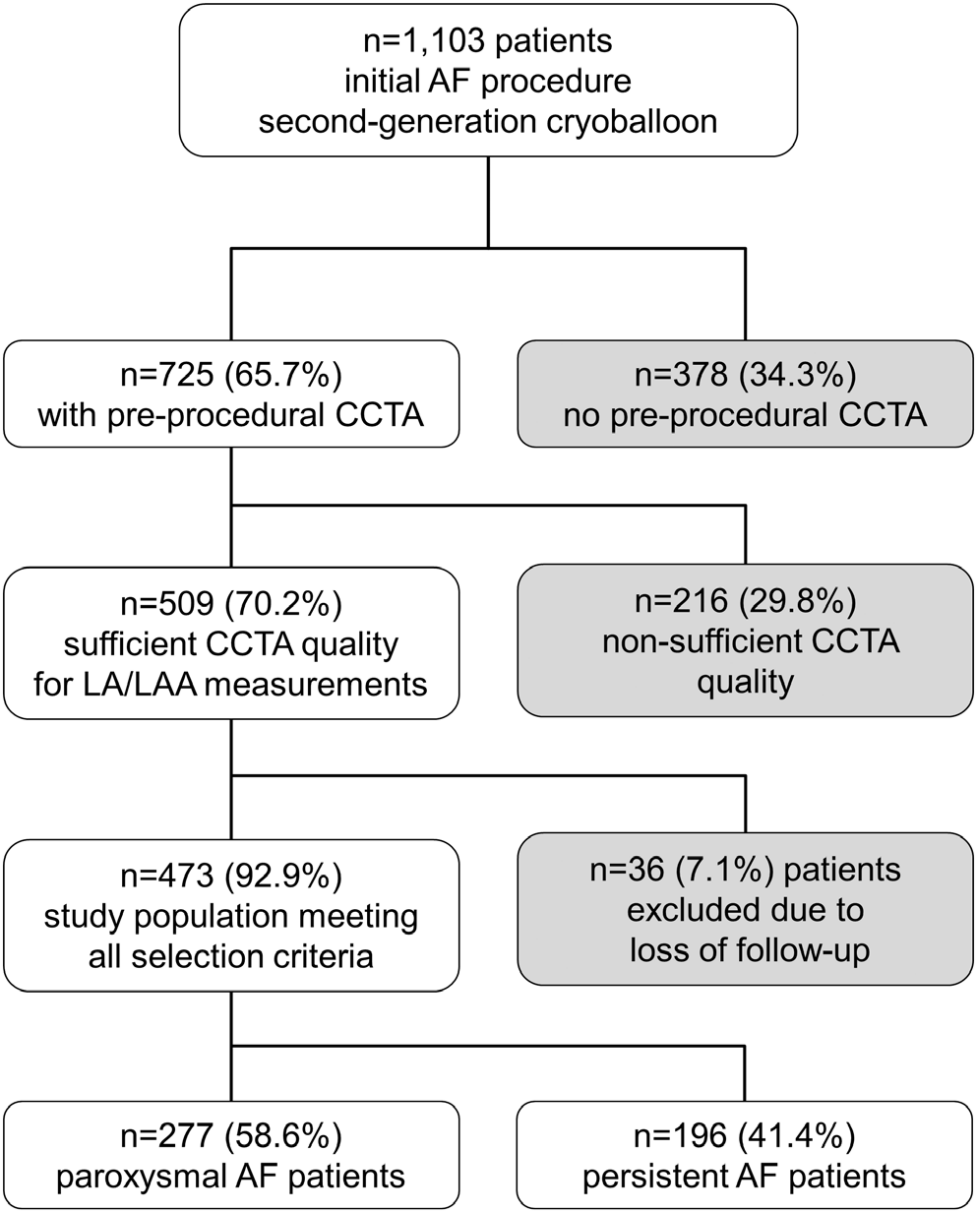
Selection criteria. This flow chart explains the selection protocol of the study population. The top box shows the number of all patients included at the beginning. Each branching demonstrates one step of selection. CCTA, cardiac computed tomography angiography.

Between May 2012 and September 2016, 1100 and three consecutive PAF and persAF patients underwent CBA as the initial ablation procedure for symptomatic AF. Preprocedural CCTA was conducted in 725 (65.7%) patients, and in 473 (65.2%) of patients, CCTA images were of sufficient quality for measuring. All patients received complete electrical isolation of PV. Mean age was 66.3 ± 9.5 years and 189 (40%) patients were females. Based on their AF types, patients suffering from PAF were designated to Group A, and those with persAF were assigned to Group B, with 277 (58.6%) patients categorized as PAF and 196 (41.4%) classified as persAF, respectively. A flow chart explaining the selection criteria is depicted in Figure 3.

### 3.2 Comparison of baseline characteristics

The comparison of baseline characteristics between patients with persAF (Group B) and those with PAF (Group A) is presented in Table 1. The primary differences observed were that Group B had a higher proportion of patients with valve disease of grade ≥ II (2.5% vs. 9.2%, *p* = .003), specifically mitral regurgitation of grade ≥ II (1.8% vs. 6.6%, *p* = .01), larger left atrial diameters (42 [7] mm vs. 45 [9] mm, *p* < .001), and lower left ventricular ejection fractions (LVEF) (60 [2] % vs. 55 [5] %, *p* < .001), as measured by transthoracic echocardiography (TTE). Other clinical factors, such as the presence of hypertensive heart disease (19.2% vs. 26.0%, *p* = .09), underlying cardiomyopathy (2.6% vs. 4.6%, *p* = .30), or prior myocardial infarction (1.5% vs. 2.1%, *p* = .73), did not show any significant differences between the two groups. Furthermore, anatomical variations in the PVs, including common ostia (20.6% vs. 22.6%, *p* = .65) and accessory PVs (17.3% vs. 17.9%, *p* = .90), were equally distributed.

**TABLE 1:** Baseline characteristics.

	Total patients *n* = 473	ParoxysmalAF *n* = 277	PersistentAF *n* = 196	*p*‐value
Age, years	66.3 ± 9.5	66.7 ± 9.5	65.7 ± 9.5	.24
Females	189 (40.0)	120 (43.3)	69 (35.2)	.09
BMI, kg/m^2^	26.5 ± 6.7	26.5 ± 4.9	26.4 ± 4.3	.90
Coronary heart disease	68 (14.4)	39 (14.1)	29 (14.8)	.89
Valve disease ≥ II^a^	25 (5.3)	7 (2.5)	18 (9.2)	.**003**
Mitral regurgitation ≥ II^a^	18 (3.8)	5 (1.8)	13 (6.6)	.**01**
LA diameter, mm^a^	43 [8]	42 [7]	45 [9]	**<.001**
Ejection fraction, %	60 [5]	60 [2]	55 [5]	**<.001**
Hypertension	309 (66.2)	179 (66.1)	130 (66.3)	1.00
Hypertensive heart disease	103 (21.8)	52 (19.2)	51 (26.0)	.09
Cardiomyopathy	16 (3.4)	7 (2.6)	9 (4.6)	.30
Prior MI	8 (1.7)	4 (1.5)	4 (2.1)	.73
Left common ostium^b^	101 (21.4)	57 (20.6)	44 (22.6)	.65
Accessory veins^b^	83 (17.5)	48 (17.3)	35 (17.9)	.90

*Note*: *n* (%), mean ± SD, or Median [IQR]. Statistically significant values are highlighted in bold.

Abbreviations: BMI, body mass index; LA, left atrial; MI, myocardial infarction.

^a^ Measured by transthoracic echocardiography.

^b^ Determined by cardiac computed tomography.

### 3.3 Differences in CCTA‐derived LA and LAA parameters

In terms of the LA and LAA measurement data obtained, Group B displayed an overall increase in dimensions. Detailed information is presented in Table 2. The most significant differences observed were the larger LA volumes (108 [34] mL vs. 125 [45] mL, *p* < .001), LAVI (57 [20] mL/m^2^ vs. 67 [19] mL/m^2^, *p* < .001), and LAA volumes (8.3 [5] mL vs. 9.2 [5] mL, *p* = .005), LAAVI (4.3 [3] mL/m^2^ vs. 4.7 [2] mL/m^2^, *p* = .019), as well as their corresponding parameters. These include the area of the posterior left atrial wall (1172 [334] mm^2^ vs. 1276 [398] mm^2^, *p* < .001), the septum orifice distance (57 [7] mm vs. 58 [7] mm, *p* < .001), the LA depth (37 [7] mm vs. 40 [8] mm, *p* < .001), the distance from the mitral valve annulus to the left atrial roof (68 [7] mm vs. 70 [9] mm, *p* < .001), and the LAA ostial area (325 [163] mm^2^ vs. 353 [155] mm^2^, *p* = .01). The PV ostial dimensions were significantly greater in Group B compared to Group A.

**TABLE 2:** CCTA derived measurement data.

	Total patients *n* = 473	Paroxysmal AF *n* = 277	Persistent AF *n* = 196	*p*‐value
LA dimensions				
LA volume, mL	115 [38]	108 [34]	125 [45]	**<.001**
LAVI, mL/m^2^	60 [21]	57 [20]	67 [19]	**<.001**
Distance of MVA—LA roof, mm	69 [9]	68 [7]	70 [9]	**<.001**
Septum orifice distance, mm	58 [7]	57 [7]	58 [7]	**<.001**
Roof top line, mm	35 [10]	35 [8]	37 [11]	**<.001**
Roof bottom line, mm	42 [9]	41 [9]	43 [10]	**<.001**
Posterior wall box height, mm	32 [6]	31 [6]	33 [6]	**<.001**
Posterior wall box area, mm^2^	1204 [391]	1172 [334]	1276 [398]	**<.001**
LA depth, mm	38 [8]	37 [7]	40 [8]	**<.001**
Max. ostial diam. LSPV, mm	21 [4]	21 [4]	21 [5]	.**04**
Min. ostial diam. LSPV, mm	14 [4]	14 [4]	15 [5]	.**04**
Area of LSPV, mm^2^	212 [135]	212 [112]	212 [174]	.17
Max. ostial diam. LIPV, mm	17 [3]	17 [3]	18 [4]	.**002**
Min. ostial diam. LIPV, mm	13 [4]	13 [4]	14 [4]	.**002**
Area of LIPV, mm^2^	155 [126]	151 [108]	174 [151]	.**05**
Max. ostial diam. RIPV, mm	18 [4]	18 [4]	19 [4]	.**001**
Min. ostial diam. RIPV, mm	15 [4]	15 [4]	16 [4]	.**03**
Area of RIPV, mm^2^	214 [96]	200 [96]	227 [101]	.**006**
Max. ostial diam. RSPV, mm	21 [5]	21 [5]	21 [4]	.09
Min. ostial diam. RSPV, mm	17 [5]	16 [4]	17 [4]	.**01**
Area of RSPV, mm^2^	276 [125]	267 [120]	294 [111]	.**02**
Max. ostial diam. LCO, mm	30 [7] *n* = 101	29 [5] *n* = 57	32 [7] *n* = 44	.**002**
Min. ostial diam. LCO, mm	17 [6] *n* = 101	16 [6] *n* = 57	19 [7] *n* = 44	.15
Area of LCO, mm^2^	388 [217] *n* = 101	364 [146] *n* = 57	487 [243] *n* = 44	.**03**
LAA dimensions				
LAA volume, mL	8.7 [5]	8.3 [5]	9.2 [5]	.**005**
LAAVI, mL/m^2^	4.6 [2]	4.3 [3]	4.7 [2]	.**019**
LAA length, mm	44 [10]	44 [11]	45 [11]	.63
Distance to the first bend, mm	12 [4]	12 [4]	12 [4]	.42
Area of LAA ostium, mm^2^	339 [162]	325 [163]	353 [155]	.**01**
Max. diam. of LAA ostium, mm	25 [6]	24 [6]	25 [5]	.**01**
Min. diam. of LAA ostium, mm	18 [5]	17 [5]	18 [5]	.**03**
Max. LAA width, mm	37 [9]	37 [9]	37 [8]	.30
Max. LAA height, mm	27 [8]	27 [8]	27 [9]	.54
Max. LAA depth, mm	40 [12]	40 [13]	40 [11]	.38

*Note*: Mean ± SD, or median [IQR]. Statistically significant values are highlighted in bold.

Abbreviations: diam., diameter; LA, left atrium; LAA, left atrial appendage; LAAVI, left atrial appendage volume index; LAVI, left atrial volume index; LCO, left common ostium; LIPV, left inferior pulmonary vein; LSPV: left superior pulmonary vein; MVA, mitral valve anulus; RIPV, left inferior pulmonary vein; RSPV, right superior pulmonary vein.

### 3.4 Differences in LAA morphology

The overall distribution of LAA morphology was as follows: “windsock” (51%), “chicken‐wing” (20%), “cauliflower” (15%), and “cactus” (13%). However, there were no significant differences observed between PAF or persAF patients, as the distribution of LAA morphology was similar in both groups: “windsock” (*p* = .78), “chicken‐wing” (*p* = .64), “cauliflower” (*p* = 1.00), and “cactus” (*p* = .26). The respective distribution is presented in Figure 2 and Table 3.

**TABLE 3:** LAA morphological distribution.

	Total patients (*n* = 473)	Paroxysmal AF (*n* = 277)	Persistent AF (*n* = 196)	*p*‐value
Chicken‐wing	98 (20.7)	55 (19.9)	43 (21.9)	.64
Windsock	244 (51.6)	141 (50.9)	103 (52.6)	.78
Cauliflower	72 (15.2)	42 (15.2)	30 (15.3)	1.00
Cactus	59 (12.5)	39 (14.1)	20 (10.2)	.26

*Note*: *n* (%).

## 4 DISCUSSION

To the best of our knowledge, this is the first study that shows that LAA morphology is not associated with the type of AF in patients undergoing initial PVI by means of CBA. Therefore, general preprocedural LAA analysis via complex imaging modalities such as CCTA or magnetic resonance imaging (MRI) seems to be redundant in the context of planning AF ablation procedures for persAF patients. Patients with persAF more frequently had underlying cardiac diseases such as valvular heart diseases and a reduced LVEF. PersAF patients showed overall enlarged LA as well as LAA features as compared to PAF patients; however, there was a highly relevant difference regarding the statistical significance level. The impact of the underlying AF type seems to be more relevant in LA enlargement than in LAA enlargement.

### 4.1 Association of LAA morphology and AF type

In this study, an equal distribution of LAA morphology was observed in both PAF and persAF patients, suggesting that LAA morphology is not linked to the underlying type of AF. This finding is in line with a previously published multicenter study by di Biase et al.^8^ In contrast, Lee et al. reported a higher prevalence of chicken wing and cactus LAA types in PAF patients.^9^ This disparity in LAA classification can be attributed to the subjective and difficult‐to‐standardize nature of LAA morphology assessment. Therefore, these findings may be subject to investigator bias to some extent. In the present study, a blinded review process was utilized to mitigate potential bias during the classification of LAA morphology types.

Nevertheless, the results of this study suggest that LAA morphology plays a relatively insignificant role in the preprocedural evaluation for AF ablation, as individual LAA morphology cannot be considered a potential risk factor for progressive AF types. Consequently, stratification based on LAA morphology may not be relevant when considering ablation strategies beyond PVI.

However, it is noteworthy that while LAA morphology does not directly influence AF type, certain shapes, such as the chicken‐wing type, have been associated with a lower risk of stroke, while more complex shapes like windsock or cauliflower may predispose patients to thrombus formation.^8^, ^9^, ^10^ These studies suggest that LAA morphology could have implications for stroke risk stratification.

### 4.2 Potential implications for ablation strategies in persistent AF

This study revealed that patients with persAF exhibit larger LA dimensions and PV ostial parameters compared to those with PAF. Literature suggests that patients who undergo a second ablation often experience PV reconnection rather than the development of new arrhythmogenic foci, particularly in those with persAF undergoing RFA.^11^ Although the current study does not establish a direct association between larger PVs and PV reconnection, studies have proposed that larger PVs with greater perimeters provide more opportunities for reconnection or that they may exhibit histological and electrophysiological abnormalities that predispose them to reconnection.^12^, ^13^, ^14^ Therefore, achieving durable electrical isolation remains crucial for improving AF recurrence rates.

CBA has demonstrated higher rates of durable PV isolation compared to point‐by‐point RFA.^15^ Furthermore, newer technologies such as the POLARx FIT® cryoballoon (Boston Scientific, Marlborough, MA, USA), which offers adjustable balloon sizes (28 or 31 mm), and the first single‐shot pulsed field ablation catheter, Farawave® (Boston Scientific, Marlborough, MA, USA), which comes in catheter sizes of 31 and 35 mm, show promising results in minimizing PV reconnection.^16^, ^17^

Overall, the identification of larger PV ostia in persAF may hold significant implications, especially as new catheter designs with adjustable diameters are evaluated for use in this patient population.

### 4.3 Empirical LAA isolation and other approaches beyond PVI

This study, comparing CCTA‐derived measurements in patients with PAF and persAF undergoing CBA, indicates that progressive AF types are linked to an enlargement of both the LA and the LAA dimensions. These findings are consistent with previous studies by Simon et al. and Amin et al., which evaluated LA and LAA features and their impact on the recurrence rate of patients after undergoing RFA.^18^, ^19^ These observations can be attributed to AF‐related electroanatomic remodeling, resulting from altered expression and/or function of cardiac ion channels, which promotes the development of functional re‐entry substrates and contributes to persAF.^20^ As AF progresses, atrial substrate becomes more complex, and AF recurrences occur despite electrically disconnected PVs. Complex ablation strategies beyond PVI were suggested, such as empirical lines, substrate ablation, isolation of the superior vena cava, posterior box isolation, the ligament of Marshall, rotors, complex fractionated electrograms ablation, or empirical LAA isolation.^2^, ^21^

It is important to note that in this imaging‐based anatomical study, the difference in the level of statistical significance regarding LA and LAA measurements indicates that persAF has a stronger association to the LA size as compared to the LAA dimensions. However, LAA might be a potential target for AF ablation as it could serve as a source of extra‐PV trigger of AF. Interestingly, there are contradictory data regarding LAA triggers of AF in the literature: A recent study by Al Rahawi et al. showed a very low incidence (0.3%) of true LAA triggers in patients undergoing catheter ablation.^22^ On the contrary, other studies suggest that up to 20% of extra PV triggers originate from the LAA, and empirical LAA isolation has been proposed as a potential treatment strategy.^23^ However, LAA isolation results in electromechanical dissociation from the LA, which greatly increases the risk of LAA thrombus formation and thromboembolism, and it should always be accompanied by a subsequent LAA closure device^24^ with its own challenges and limitations, such as periprocedural pericardial effusion, device thrombus formation, or leakage. Interestingly, the CCTA data in this study showed a notably larger LA posterior wall box area in patients with persAF compared to those with PAF. This observation suggests catheter ablation aiming at electrical posterior wall isolation may pose greater challenges in persAF due to the potential need for longer ablation lines and the risk of gap formation.

The existing guidelines recommend PVI to be the cornerstone of catheter ablation for persAF.^2^ From the anatomical perspective of this study, the primary focus in patients with recurrent persAF and durably isolated PVs should be on strategies targeting the LA rather than the LAA in most patients. Moreover, the present data do not support ablation strategies tailored according to LAA morphology.

### 4.4 CCTA prior to pulmonary vein isolation

Although preprocedural cardiac CCTA of the LA is often conducted to guide catheter ablation of AF, it remains uncertain whether this imaging is associated with improvements in efficiency, efficacy, and safety.^25^ On the one hand, preprocedural CCTA can provide valuable anatomical information regarding the LA, PVs, and LAA, and it can rule out underlying coronary artery disease or intracardiac thrombi.^26^ On the other hand, CCTA has three major downsides: radiation exposure, the use of contrast media containing iodine, which can impair kidney function or thyroid hormone balance in otherwise healthy patients, and the availability of this large‐scale imaging technology.

The recently published DECAAF II trial and the ALICIA trial indicated that PVI plus preprocedural magnetic resonance imaging‐guided fibrosis ablation did not provide additional benefits compared to a PVI‐only strategy in patients with persistent AF.^27^, ^28^ In fact, more periprocedural cerebrovascular events were observed in the group with fibrosis ablation in addition to PVI. Moreover, precise measurements of LA anatomy can also be determined intraprocedurally via LA angiography, 3D electroanatomic mapping of the LA, or intracardiac echocardiography.

Consequently, TTE and transesophageal echocardiography (TEE) are typically preferred as primary preprocedural imaging modalities. Their advantages include low cost, widespread availability, and the ability to provide sufficient measurements without the need for radiation or contrast agents. However, regarding precise LAA measurements or LA substrate analysis, echocardiography offers only limited options.

In summary, the routine usage of preprocedural imaging modalities such as CCTA or MRI should be critically assessed, as their disadvantages may outweigh the benefits. In most cases, echocardiography should remain the modality of choice. However, in complex AF patients undergoing secondary ablation procedures, precise anatomical analysis via CCTA or MRI could offer valuable insights in studies following appropriate protocols, particularly when empirical strategies beyond PVI are being considered. Additionally, imaging‐based assessment of LAA morphology is crucial for planning combined endocardial and pericardial LAA occlusion procedures.^29^

## 5 LIMITATIONS

There are several notable limitations to consider in this study. It is a single‐center, prospective study with a retrospective evaluation, which inherently carries biases associated with this design. Not all patients underwent CCTA before the AF ablation procedure, so the data is not fully consecutive. Additionally, image quality varied, making data assessment more challenging in some cases than others. Although CCTA measurements were generally performed in fasting patients, variations in volume loading could still influence the measurements of the left atrial and left atrial appendage volume. Although four experts determined and validated the LAA morphology classification, the criteria remain somewhat subjective and imprecise, leading to variations in the distributions reported in current literature. This anatomical imaging study focuses on comparing left atrial characteristics between patients with PAF and persAF. It does not include outcome data on stroke risk or AF recurrence, information on extra‐pulmonary vein AF triggers, such as those from the LAA, or an analysis of PVI durability following CBA. Additionally, post‐ablation 3D voltage mapping was not utilized as part of the study protocol. While voltage mapping could provide valuable insights into the extent of electrical isolation and its potential correlation with anatomical differences observed in the CCTA, it was not part of the approach in this study. Therefore, data on the isolation areas or postablation voltage patterns are not available. Last but not least, data on PV reconnection were not collected, and therefore, the hypothetical implications mentioned above should be verified in future trials.

## 6 CONCLUSION

In patients undergoing initial PVI via CBA, there is no association between LAA morphology and the type of AF, indicating that LAA morphology may not be a significant factor in planning ablation strategies.

Patients with persAF more frequently presented with reduced LVEF and heart valve diseases. Additionally, they exhibited larger dimensions of the LA, PV, posterior wall box area, and LAA compared to those with PAF. Future research should investigate the influence of personalized ablation approaches, considering individual patient characteristics, to optimize treatment results for patients with different forms of AF. Preprocedural CCTA or MRI scans might reveal crucial insights for complex AF patients if ablation strategies beyond PVI are investigated in well‐planned studies.

## FUNDING INFORMATION

A research agreement between the local institution and Abbott Medical exists, and the clinical software of EnSite Precision™ (Abbott Medical GmbH, Eschborn, Germany) was provided for the retrospective reconstruction and postprocessing of the CCTA images.

## CONFLICT OF INTEREST STATEMENT

Dr. Straube received honoraria for lectures from Medtronic, Boston Scientific, Philips, Bristol‐Myers‐Squibb, and Astra Zeneca outside the submitted work. Dr. Dorwarth reports honoraria for lectures from Medtronic Inc., Bristol‐Myers‐Squibb, and Astra Zeneca, outside the submitted work. Dr. Hoffmann is head of the department; the department received compensation for participation in clinical research trials outside the submitted work from Abbott, Bayer, Biotronik, Boehringer Ingelheim, Edwards, Elixier, Medtronic, and Stentys. Dr. Hartl received honoraria for lectures from Bristol Myers Squibb outside the submitted word. Dres. Brueck, Olbrich, Pongratz, Riess, Tesche, and Wankerl have nothing declared.

## ETHICAL APPROVAL

All procedures followed were in accordance with the ethical standards of the responsible committee on human experimentation (institutional and national).

## INFORMED CONSENT

Informed consent was obtained from all patients to participate prior to the procedure. Data were entered in the institutional database.

## CLINICAL TRIAL REGISTRATION

The institutional registry study was approved by the Ethics Committee of the Bavarian State Medical Association (Reference number: 7/11140).

## PERMISSION TO REPRODUCE MATERIAL FROM OTHER SOURCES

Not applicable.

## Data Availability

The data underlying this article will be shared on reasonable request to the corresponding authors.
